# Essential terminology and considerations for validation of non-targeted methods

**DOI:** 10.1016/j.fochx.2022.100538

**Published:** 2022-12-10

**Authors:** Kapil Nichani, Steffen Uhlig, Manfred Stoyke, Sabine Kemmlein, Franz Ulberth, Ilka Haase, Maik Döring, Stephan G Walch, Petra Gowik

**Affiliations:** a QuoData GmbH, Prellerstr. 14, 01309 Dresden, Germany; b Institute of Nutritional Sciences, University of Potsdam, Arthur-Scheunert Allee 114-116, 14558 Nuthetal, Germany; c QuoData GmbH, Fabeckstr. 43, 14195 Berlin, Germany; d Bundesamt für Verbraucherschutz und Lebensmittelsicherheit (BVL), Diedersdorfer Weg 1, 12277 Berlin, Germany; e European Commission, Joint Research Centre, Retieseweg 111, 2440 Geel, Belgium; f Max Rubner-Institut (MRI) - Bundesforschungsinstitut für Ernährung und Lebensmittel, Nationales Referenzzentrum für authentische Lebensmittel, E-C-Baumannstr. 20, 95236 Kulmbach, Germany; g Chemisches und Veterinäruntersuchungsamt (CVUA) Karlsruhe, Weißenburger Str. 3, 76187 Karlsruhe, Germany

**Keywords:** Non-targeted methods, Method validation, Food fraud, Food authenticity, Mass spectrometry, NMR, Spectroscopy, NGS

## Abstract

Through their suggestive name, non-targeted methods (NTMs) do not aim at a predefined “needle in the haystack.” Instead, they exploit all the constituents of the haystack. This new type of analytical method is increasingly finding applications in food and feed testing. However, the concepts, terms, and considerations related to this burgeoning field of analytical testing need to be propagated for the benefit of those associated with academic research, commercial development, or official control. This paper addresses frequently asked questions regarding terminology in connection with NTMs. The widespread development and adoption of these methods also necessitate the need to develop innovative approaches for NTM validation, i.e., evaluating the performance characteristics of a method to determine if it is fit-for-purpose. This work aims to provide a roadmap for approaching NTM validation. In doing so, the paper deliberates on the different considerations that influence the approach to validation and provides suggestions therefor.

## Highlights

• Increasing applications of non-targeted methods (NTMs) in the detection of food fraud and determination of food authenticity.
• Understanding the expanding set of terminologies and notations around NTMs for widespread adoption.
• Considerations on how to validate NTMs so that they can be used in the routine.

## 1 Introduction

A new type of analytical method, known as “non-targeted methods” (NTMs), has emerged as a powerful technique for tackling problems in fields such as food authenticity (including food fraud) (Ulberth, 2020), food quality (Böhme, Karola, Calo-Mata et al., 2018), food safety (Medina et al., 2019), water monitoring (Hollender et al., 2017), microbial species subtyping (Singhal et al., 2015), among others. These new methods are described in a growing body of literature, and some are already being used in routine testing and monitoring (Monakova et al., 2014, PerkinElmer, n.d., Solovyev et al., 2021). Many different terms are used to refer to NTMs in the literature, such as “untargeted method,” “non-target testing,” “non-target approaches,” and “fingerprinting methods,” among others (Ballin & Laursen, 2019). In this paper, only the term “non-targeted methods” will be used.

NTMs combine the superiority of high-resolution analytical measurement instruments with advances in chemometrics and machine learning algorithms. Acquired measurements typically consist of large arrays of values, which are sometimes referred to as the “fingerprint” of the sample under examination. Owing to the immense potential of these methods, the past decade has seen their rapid development and adoption by researchers and laboratories, especially in connection with food authenticity. As a result, there is a pressing need to devise ways to ensure the reliability of the obtained results. The key to doing so is providing objective evidence that NTMs are fit for their intended purpose via method validation (MV) studies. In such studies, method performance characteristics are obtained and communicated in such a way as to allow informed decisions by producers, consumers, official control agencies, and regulators alike (Magnusson and Örnemark, 2014, Thompson et al., 2002). European Union (EU) Official Controls Regulation requires official food control laboratories to apply, when available, standardized methods, i.e., methods validated in a multi-lab validation study (MLV) (EU Controls Regulation: REGULATION (EU) 2017/625 OF THE EUROPEAN PARLIAMENT AND OF THE COUNCIL of 15 March 2017 on Official Controls and Other Official Activities Performed to Ensure the Application of Food and Feed Law, Rules on Animal Health, and Welfare, 2017). If a standardized method is not available, methods validated in a single-lab validation study (SLV) should be applied. In such cases, the comparability of results between official food control laboratories and commercial laboratories doing the counter analysis on behalf of the business operators becomes the challenge. Several researchers and experts have drawn attention to the paucity of internationally accepted validation protocols for NTMs (Cavanna et al., 2018, McGrath et al., 2018, Riedl et al., 2015). Thus, much has to be addressed regarding how to perform NTM validation (Creydt and Fischer, 2020a, Esslinger et al., 2014, Locatelli et al., 2017, Riedl et al., 2015).

To this end, the purpose of this paper is twofold. First, to review and describe the terminologies in connection with NTMs. The burgeoning field of NTMs has been accompanied by an expanding set of terminologies. These terms are related to the new analytical technologies, the machinations of artificial intelligence methods, information systems infrastructures, and statistical decision theory. Hence, there is considerable room for misinterpretation of what different experts might be conveying. Second, it aims to investigate the factors that make devising an NTM validation protocol challenging. In doing so, it highlights the points of contention surrounding validation choices. This work proposes considerations for how NTM validation can be implemented in practice. Altogether, this work aims to add to the limited body of work that is currently available. This will eventually help researchers in the scientific community, officials at control agencies, and experts in drafting relevant guidelines, protocols, or standards.

The paper is organized as follows: Section 2 describes the terminology relating to NTMs. The novelty here is that this construction of terminology, although opinionated, can provide a common basis for any dialogue on the topic of NTMs. Section 3 provides a general discussion of the basics of method validation. The following section then reviews the concepts that are currently available for NTM validation. Given the current understanding of NTMs and their validation, these three sections segue into specific proposals for the NTM validation procedure, detailed in 5, 6, respectively. The validation scheme involving quantitative scores proposed in Section 6 has not been reported previously, to the best of our knowledge, and is one of this paper’s most important contributions. Next, we discuss the validation sample requirements in Section 7. And finally, we lay out the stages for NTM development and validation and how we see them differing from the traditional way. Previously inaccessible, this section describes the procedure for collaborative method development in NTMs and its considerations for validation.

## 2 Describing NTMs and the terminology around it

Devising MV concepts for NTMs such that it can bring under its ambit all the different methods that are available currently and future methods is a challenging task, to say the least. A good place to start is by asking what constitutes an NTM. In the following, eight questions are addressed in order to gradually elucidate the concept of NTM. Instead of providing an unambiguous definition for NTM, the discussions arising in connection with these questions will shed light on the manifold aspects of NTMs.

### 2.1 What are the components of an NTM?

**Fig. 1:**
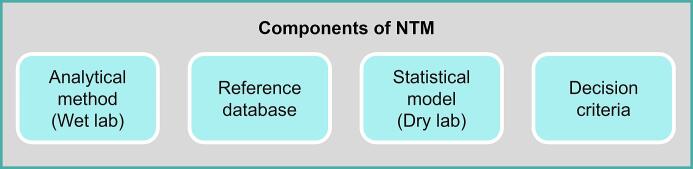
Components of a non-targeted method.

Fig. 1 shows the general components of an NTM. All steps involved in the NTM until the analytical measurements, which are performed on a lab bench, are collectively referred to as “wet lab” procedures. Some manner of chemometric / statistical / machine learning / artificial intelligence model is then responsible for parsing this multi-dimensional dataset. These steps are referred to as the “dry lab” procedures. The copious amounts of measurement data are saved, processed, and retrieved by means of electronic or reference databases. An NTM uses several features obtained by measurements (in the wet lab) in combination with data analytics (in the dry lab using a reference database) to authenticate a food product (product characteristic). Food authenticity testing can be done in relation to the species, origin, production or processing system, purity, etc. Ultimately, the final decision regarding authenticity is usually based on a set of criteria.

The above terminology will prove very useful in determining what can (and cannot) be considered an NTM. It should be emphasized that the analytical method used to obtain measurements for building the reference database must be validated regarding its analytical performance in such a way (e.g., SLV) that it is poised for eventual standardization (further elaborated in Section 8). This is important to ensure that the data basis in the reference database is not rendered unusable, leaving any ensuing NTM development unsuitable.

### 2.2 Are NTMs associated with a particular measurement platform or instrument or analytical technology?

In its simplest sense, a “measurement platform” refers to the analytical measurement procedure. The term “platform” encompasses the ensemble of instruments and equipment involved in the wet lab, which is not only highly complex and expensive but also of considerable size. Having said that, measurement instruments can also be portable benchtop or handheld devices. The different wet-lab analytical technologies that are typically part of NTMs are as follows: Methods involving chromatographic separation (like gas or liquid chromatography) are mostly, but not exclusively, followed by (high-resolution) mass spectrometry (Creydt & Fischer, 2020b). Alternatively, these can be other spectra-generating methods such as nuclear magnetic resonance (NMR) (Sobolev et al., 2019), Fourier transform infrared (FTIR) (Segelke et al., 2020), near-infrared (NIR) (Grassi et al., 2018), or Raman spectroscopy (Xu et al., 2020). In practice, NTMs are not specific to any instrument, measurement platform, or analytical technology. Moreover, a combination of several analytical measurement platforms can also be used simultaneously in connection with an NTM.

We believe that NTMs involve not only spectra, chromatograms, or identified and quantified chemical entities (elements, fatty acids, etc.) as measured signals from the instruments but also nucleotide sequences. This can be the case in metabarcoding methods involving next-generation sequencing (NGS) technologies applied in order to identify several species or taxa simultaneously (Haynes et al., 2019). Whether these methods are NTMs can be argued with the reasoning that a genomic region (barcode) is targeted by a defined primer pair. However, this primer pair needs to fit universally to the selected genetic region in all organisms of interest (e.g., all land vertebrates), allowing non-targeted species identification via multiple parallel sequencing and subsequent assignment of the sequences by database comparison.

### 2.3 Are databases required for NTMs?

The short answer is: Yes! In the generic sense, large amounts of empirical data are acquired to define the sample populations (classes), e.g., wine from France and wine from Germany. It should be noted that, depending on the context, reference databases will address different types of classification, for instance, geographic origin or production process (e.g., organic versus conventional).

However, there are further aspects. Indeed, like much of the terminology used in connection with NTMs, the notion and implication of the term “database” can be diverse. For instance, “database” may refer to the entire technology behind the storage and management of data (in the cloud or locally). Alternatively, “database” could refer to the collection of data stored in it. It is not always required to have a technical database implementation for NTMs.

In any case, empirical data plays an important role in NTMs, and one should be aware of the context in which the term “database” is used. The generation of data from well-defined samples is mandatory, and together with meta-data capturing the related traceability information, these are the most common types of databases used by NTMs. Usually, the greater the amount of training data available, the more accurate the model is. The reference database can be used to “train” supervised machine learning algorithms. Alternatively, the measured data can be compared against the reference sample data using non-supervised approaches, e.g., similarity metrics or correlation coefficients.

### 2.4 Do NTMs always help answer a yes or no question?

Broadly, food authenticity testing aims to investigate whether claims made for a particular food item are correct (e.g., regarding the geographic origin, plant/animal species, ingredients, process). Typically, the classification consists of matching the sample to classes as defined in a reference database. The output of the dry lab procedure furnishes quantitative values such as probabilities (of belonging to a given class), referred to as “decision scores.” The sample is then assigned to a particular class (say, class α) if their score is below a given univariate decision threshold (or decision limit). Conversely, a sample is assigned to the other class (say, class β) if their score is higher than the decision threshold. Experience shows that quantitative decision scores exist for a large number of NTMs owing to the underlying chemometric or machine learning model (Alewijn et al., 2016). The outcome of the decision will undoubtedly be yes or no.

### 2.5 What is the difference between ‘fingerprinting’ and ‘profiling’, and are both NTMs?

Considering the definitions reviewed and provided by Balin et al. profiling methods fall into the type of targeted methods (Ballin & Laursen, 2019). An alternative perspective is that both fingerprinting and profiling are NTMs as both require a statistical model (dry lab) and a reference database for decision making (Fig. 1). The main difference between fingerprinting and profiling relates to the output generated by the analytical method (wet lab), in other words, whether it targets specific entities. Fingerprints of a material are electronic records (e.g., whole or part of chromatograms or spectra) produced by an instrument without further information regarding the identities or quantities of entities represented by the record, whereas quantity values of defined entities constitute the profile of a material (e.g., elements, fatty acids, sugars, etc.) (Ballabio et al., 2018, Danezis et al., 2017, Sørensen et al., 2016). However, the profile itself does not allow us to decide on the authenticity of the material but is used as the input of a multivariate decision model. Quite often, fingerprinting methods are converted into profiling methods by attempting to identify the most relevant variables for discrimination. Usually, this variable reduction reduces noise and guards against model overfitting, but the biggest advantage is that the resulting targeted, profiling methods are independent of the measurement platform through calibration with reference materials.

### 2.6 Is ‘suspect screening’ also a type of NTM?

Suspect screening workflows are widely used in food, environmental, and forensic chemistry (Ballin and Laursen, 2019, Hollender et al., 2017). In these types of pipelines, a large list of suspect compounds (n ≫ 1) is checked for presence or absence (Hollender et al., 2017). In food analysis, such methods are mainly used for food safety questions (e.g., pesticide screening) and are less known for authenticity questions. Therefore, in this paper, such workflows may not be considered NTMs, *stricto sensu*, but do make use of the information content from the multi-dimensional measurement data.

### 2.7 What is the role of calibration in an NTM?

The IUPAC definition of calibration “*the set of operations which establish, under specified conditions, the relationship between values indicated by the analytical instrument and the corresponding known value of an analyte*” is in principle applicable to NTMs if the state of the material (authentic or non-authentic) is regarded as the quantity value to be determined (IUPAC Compendium of Chemical Terminology, 2014). However, in contrast to methods where the quantity of a targeted analyte (measurand) is estimated via a univariate calibration function (e.g., linear, or quadratic regression), NTMs use multivariate models for deciding whether the sample is authentic. Such models need to be calibrated as well, but to avoid confusion, the term “training” is frequently used instead, and the samples used for setting up the model are called “training samples” or “training set.”

### 2.8 What is the role of quality control materials in an NTM?

As with any other analytical method, quality control is a prerequisite for producing valid results, requiring the availability of quality control materials (QCMs). The main function of QCMs is to monitor the accuracy (precision, trueness) and stability of the analytical method during its application. (Certified) reference materials (C)RMs) are key for establishing metrological traceability and can also be used for creating quality control charts to keep a targeted method of analysis under statistical control over time. (C)RMs for NTMs are rare but a few exist, e.g., NIST SRM 1950 Metabolites in Human Plasma, which has values assigned for approximately 100 analytes. Anhydrous butter fat (BCR-519) and cocoa butter (IRMM 801) have been made available for checking the authenticity (purity) of milk fat and cocoa butter by triglyceride analysis in combination with multivariate statistical analysis. Other types of commercially available quality control materials include, e.g., meat from different species (LGC Ltd.), or plant specimens obtained from botanical gardens.

For novel NTMs such (C)RMs do not exist in most cases (Gao et al., 2019). Pooling aliquots of reference samples used in training the decision model and including them in the analytical sequence is a frequently used quality control technique. The obtained data are not only subjected to multivariate analysis (e.g., principal component analysis and inspection of the score plot), but also to generate Shewhart charts of principal components or extracted features, etc. (Lörchner et al., 2022). Such tools are appropriate for setting up quality control in a single laboratory, but they may be insufficient if the NTM is to be used in multiple laboratories. This situation frequently requires QCMs for the normalization of data produced in different laboratories and/or by different instrument brands. A QCM obtained by pooling samples and making it available as a “normalization sample” to interested laboratories is a potential solution. However, its long-term stability as well as the effect of renewing the normalization sample on data quality once the original batch is used up, have to be considered.

## 3 Validation definitions

A fundamental matter of contention is the perception of the term “validation.” Depending on the problem, context, and application, it may have a different meaning to different stakeholders. MacNeil et al. aptly pointed out that validation, just like beauty, is in the eyes of the beholder (MacNeil et al., 2000). In the context of NTMs, validation often refers to “model validation” (dry lab) (McGrath et al., 2018, Riedl et al., 2015), which can be done using techniques such as “nested cross-validation” or “k-fold validation” (details of these techniques are described elsewhere (Filzmoser et al., 2009, Uhlig et al., 2019). Thus, it is necessary to revisit MV definitions and review what can be applicable to NTM validation.

The ISO/IEC 17025:2017 standard defines validation as the “*provision of objective evidence that a given item fulfills specified requirements, where the specified requirements are adequate for the intended use*” (ISO 17025/IEC:2017 General Requirements for the Competence of Testing and Calibration Laboratories, 2017). The Eurachem Guide on the fitness-for-purpose of analytical methods defines it as “*the process of establishing the performance characteristics and limitations of a method, and the identification of the influence which may change these characteristics and to what extent*” (Magnusson & Örnemark, 2014). In ISO 16140–1:2016, MV is defined as “*Determining the performance characteristics of a process and provide objective evidence that the performance requirements for a specified intended application are met*” (ISO 16140–1:2016, Microbiology of the Food Chain—Method Validation—Part 1: Vocabulary, 2016). Classical MV concepts involve the evaluation of method performance characteristics (Accuracy (Trueness and Precision) of Measurement Methods and Results”, Parts 1-6, International Standard ISO 5725-1, 1994, Guidelines for the Validation of Chemical Methods for the FDA Foods Program, 2019, Thompson et al., 2002). Method validation usually consists in conducting experiments in a single laboratory or in several laboratories to determine these performance characteristics. Although there may be differences in meaning between single-lab vs. in-house validation or multi-lab vs. collaborative validation, for the sake of simplicity, we will consistently use the terms single-lab and multi-lab.

Methods can be validated for more than one analyte, for different matrices, or for different instruments or platforms. If available, (C)RMs can be used to determine the precision and trueness of a method. Validation protocols may include the use of (C)RMs or matrix spiked samples to determine recovery rates, matrix blank samples to determine background levels, blanks to determine the limit of detection, and replicate analysis of a sample to determine precision. The performance characteristics for the validation of a method strongly depend on the intended use, the type of method (quantitative or qualitative), or, in the case of method extension (new analyte, new matrix, new platform, etc.), the degree to which it has been previously validated. We believe that there is unanimous agreement that the performance characteristics of a newly developed quantitative method include trueness, precision, selectivity, limit of detection, limit of quantitation, linearity (or other calibration models), working range, measurement uncertainty, ruggedness, confirmation of identity, and recovery rates. It also seems undisputed that sensitivity, selectivity, false-positive rate (FPR), and false-negative rate (FNR) are typical performance characteristics for the validation of a new qualitative method. Some of these classical experiments can be transferred to NTMs, while others are simply not available or applicable (such as the use of (C)RMs, etc.).

Many NTMs are motivated by and related to a binary decision problem (discussed in detail in the following sections). A validation procedure for such NTMs, in general, involves determining the risk of a false positive or false negative decision. In this paper, “validation procedure” and “validation approach” might be used interchangeably. The performance characteristics (or figures of merit) of non-targeted methods and the way they are estimated undoubtedly differ from the ones related to targeted methods; however, the ultimate objective remains the same, i.e., demonstrate the “fitness-for-purpose” of the method, independent of the physico-chemical principles of the analytical method, the data evaluation, etc. For the method developer, this is important to objectively demonstrate the fitness for its intended use; for the method user, it is important for quality assurance and accreditation.

## 4 Existing concepts for NTM validation

The guideline for the development and validation of non-targeted methods from the US Pharmacopeia (USP) has been a go-to resource in the absence of other harmonized guidelines or standards (US Pharmacopeia, 2019). The guideline is part of the USP Food Chemicals Codex and describes a procedure for methods used to classify samples as either adulterated (atypical) or unadulterated (typical). The USP guideline defines NTM as follows: “*A method that determines the similarity of a sample (U) to a reference standard or set (Sn). It has a binary output—the sample is atypical or typical with respect to the known sample set. The concept of non-targeted methods covers a spectrum from truly non-targeted (largely theoretical) to semi-targeted (most practical applications), but for the purposes of this paper, any broadly nonspecific adulterant detection method is treated as non-targeted, as the same principles are applicable*” (US Pharmacopeia, 2019). It is noteworthy that the prescribed procedure in the USP guideline is independent of the analytical technology or food type. This is beneficial to ensure horizontal applicability to a wide range of methods. However, the scope of the guideline remains limited to a subset of methods for food authenticity testing, namely: one-class classification methods for testing adulteration or mixing.

The recommended performance characteristics include evaluation of sensitivity for the correct identification of unacceptable samples as “atypical” and specificity for the correct identification of acceptable samples as “typical.” In other words, the sensitivity of the method is the rate of detection of adulterated or fraudulent samples, and the specificity is the rate of detection of safe or compliant food items.

The USP guideline’s appeal also comes from the fact that it takes into account method development along with single lab validation. A generic thought process is described so that sufficient method suitability is established before going to the validation stage. The performance characteristics are checked against the criteria set upfront in the applicability statement. Furthermore, the guide specifies which sample sets are necessary at each stage. In the method development stage, the mathematical model is developed using a reference dataset containing adequately represented, unadulterated (authentic) samples. The model is optimized using a test set. In the validation phase, an independent sample set comprising both typical and atypical samples is tested as unknown samples. It should be noted, however, that no guidance is provided regarding the minimum number of samples in the validation set.

As a result of reliance on a specific version of the instrumentation hardware, reference databases, and complex mathematical models, NTMs might be required to be updated and revalidated. Besides, the necessary samples in the reference database might be influenced due to environmental or anthropogenic factors, leading to drifts in the mathematical model parameters. The USP guide also mentions such scenarios and recommends monitoring the method. This needs to be addressed not only by the method developer (e.g., database maintenance), but also by the user (laboratory) in the framework of quality assurance. However, it is not clear whether institutions are willing to carry the resource burden of revalidating the method (see also Section 8).

Apart from the USP guide, standard method performance requirements (SMPRs®) for non-targeted testing (NTT) are also available from the AOAC (AOAC, 2016). At the time of writing, SMPR are available for methods testing for economically motivated adulteration in three food items, viz. extra virgin olive oil (EVOO) (AOAC, 2020b), honey (AOAC, 2020a), and bovine milk (AOAC, 2020c), and draft SMPRs are available for vanilla (AOAC, 2021b), and saffron (AOAC, 2021a). These SMPRs define the NTT method as: “*Any method generating a baseline fingerprint of the authentic material and comparing test sample fingerprints to assess differences will be considered. The final binary result identifies test samples as either authentic or potentially adulterated*” (AOAC, 2020d). Unlike the USP guide, the SMPRs do not describe generic steps to adhere to in the method development stage. But they do provide a number for the samples required to be tested in the validation stage. Furthermore, it is to be noted that the AOAC SMPRs describe procedures for single-lab validation.

Together, these NTM validation resources have several attractive features that can be used for a future harmonized NTM validation concept. For example, (i) descriptions of applicability statements such as “a non-targeted method for detecting the adulteration of honey with sugar syrups at a level >10 % with a sensitivity rate of 90 % and a specificity rate of 95 %, both with a significance level of p = 0.05″ (USP), (ii) proposed performance characteristics for binary non-targeted methods, (which are more straightforward) (USP) (iii) requirements for the number of samples needed to reach a certain level of confidence (AOAC), and (iv) importance of method monitoring and need for revalidation in case of drift (USP).

A harmonized protocol for the validation of NTM should ideally build on existing proposals and proven design principles of internationally accepted protocols. These can serve as a springboard to establish an NTM validation framework. Existing harmonization efforts are ongoing in Europe (CEN) and North America (AOAC). At the same time, they should be scrutinized for scientific validity and extended for practicability and applicability. Additional work is also required to merge philosophies and specify terminologies for the different working groups, communities, and existing documents. Furthermore, effort is required to describe the principles in more detail (nature of samples, number of replicates, number of laboratories, etc.) and data requirements (number of samples) for single-lab as well as multi-lab validation. Building on the existing concepts for NTM validation, the following section proposes four NTM validation procedures.

## 5 Extending the existing concepts to NTM validation

### 5.1 Considerations for one-class, two-class, and multi-class NTM

**Fig. 2:**
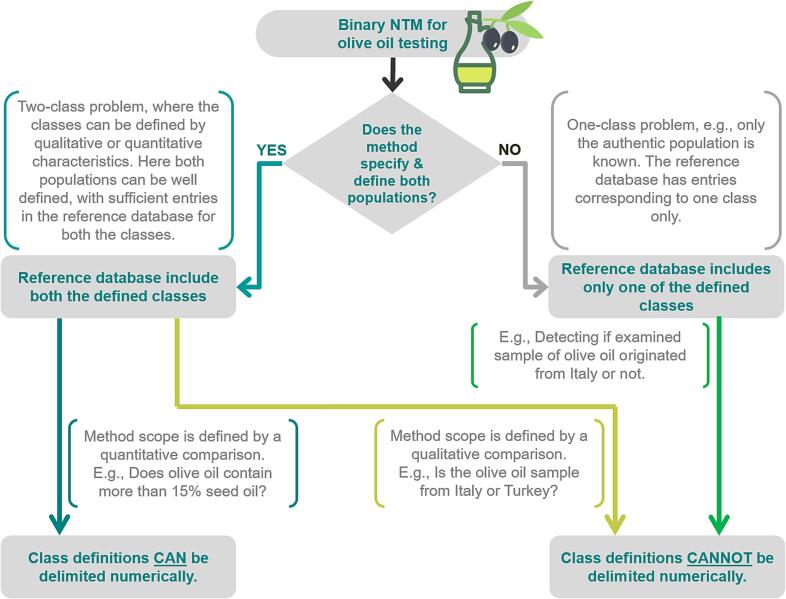
Different ways to formulate an NTM, focusing on examples around olive oil testing. The text in the white box adjoining each connecting arrow provides context and examples.

NTMs can be defined and structured in different ways. Fig. 2 serves to illustrate this perspective. Consider methods related to testing the authenticity of olive oil as a running example throughout. The first question is how to specify the scope of a binary NTM. Suppose it is a two-class problem, e.g., if the method is applied to distinguish genuine olive oil from olive oil adulterated with seed oil (e.g., sunflower oil) at some economically relevant level (e.g., 15 %). The reference database must contain entries for both classes, i.e., samples with adulteration greater than 15 % seed oil and samples with less than or equal to 15 % seed oil. By contrast, in the case of a one-class problem, only entries for one class (e.g., authentic olive oils) are required in the reference database.

The advantage of the one-class problem is that only samples corresponding to one class (e.g., authentic olive oils) are required, and it is easier for the method developer to obtain the samples. However, one-class problems do not consider sensitivity, or FNR, with respect to a specific class and only consider specificity, or FPR. One of the consequences of this is that the sensitivity of an NTM for a one-class problem is typically lower than that of a two-class problem.

One option to overcome the limitations of one-class problems from the perspective of validation would be to define a counter-class. The counter-class comprises samples that represent a reasonable approximation of samples being “non-authentic” with regard to the initial classification question. Consider the example in Fig. 2: Does the olive oil originate from Italy or not? The one class includes all possible olive oils originating from Italy. Here the counter-class (olive oils not originating from Italy) can be characterized by performing measurements on olive oil samples typically found in the market, which are from nearby countries. Defining a counter-class in this way with samples likely to be candidates for fraud can allow us to use the more efficient two-class problem validation.

### 5.2 Ways to define classes

The underlying classes can be delimited numerically, e.g., an olive oil sample belongs to the class if its seed oil content is below 15 %. More generally, the numerical demarcation can be based on adulteration level, concentration, contamination, etc. Such numerically delimited class definitions have been reported in the literature (Alamprese et al., 2016, Botelho et al., 2015, Nichani et al., 2020, Weesepoel et al., 2020).

On the other hand, if the method tests, say, whether the examined olive oil sample originates from Italy or Turkey—the classes cannot be delimited numerically. We assume that most NTMs that do not involve measures of purity (mixture or adulteration levels) involve qualitatively delimited classes. One-class problem descriptions typically involve qualitatively delimited classes. Coming back to the example of an NTM applied in order to determine whether olive oil samples originate from Italy, the two classes would be “Italy” and “not Italy.”

In addition to one- and two-class problems, NTMs are also applied in connection with multi-class classification problems (more than 2 classes). Multi-class problems can be broken down into several binary classification problems. Hence, the points discussed above can be extended to the multi-class case. Consider the example of an NTM applied to determine whether olive oil originates from Italy, Spain, or Greece. The reference database must include data for the three classes (Italy, Spain, and Greece). Such a multi-class problem can be broken down into a series of binary classification problems as follows: EU region or not; Italy or not; Spain or not; Greece or not. The conversion of multi-class problems into a series of binary classifications will likely prove useful in the validation of NTMs, particularly in the field of authenticity testing.

### 5.3 NTM validation approach

The above examples of the different types of classification problems by no means constitute an exhaustive list. Nonetheless, these examples illustrate that methods which differ, e.g., in terms of class definition or number of classes, will require different validation approaches. We now turn to an illustration of what the different validation approaches can be.

In almost all cases, the final decision step in an NTM applied in connection with a classification problem is based on a quantitative decision score calculated in the dry lab stage. This score is compared to a specified decision limit. Examples of quantitative decision scores include correlation coefficients, similarity metrics, class assignment probabilities, principal component scores, proprietary scores provided by commercial software, etc. Basing the validation on such quantitative scores (rather than on yes/no results) allows for improved method performance characterization while considerably decreasing the workload (number of samples and number of laboratories). Depending on whether classes are numerically delimited and on whether quantitative decision scores are used, 4 different validation approaches can be distinguished, (named generically as A, B, C, and D).

**Fig. 3:**
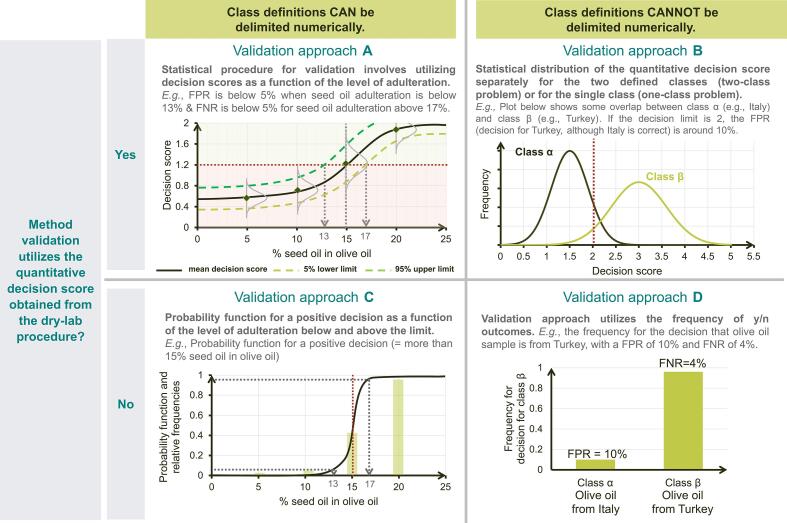
Options for validation approaches depending on whether the class definitions can be delimited numerically and whether the method validation utilizes quantitative decision scores or not.

Fig. 3 provides an overview of these 4 different approaches, illustrated on the basis of the running example for olive oil testing (Fig. 2). Please note that the illustrations in Fig. 3 do not cover the entire validation procedure. However, they provide a graphic comparison of how performance characteristics will be evaluated. Validation approach A is used when (i) the classes are delimited by the level of seed oil adulteration in olive oil and (ii) decision scores from the dry lab statistical model are used. Fig. 3 (top left) illustrates how the decision score distribution depends on the adulteration level. A decision limit of 1.2 is considered here (as an example). The two classes are delimited numerically, namely: above and below 15 % adulteration with seed oil. It can be seen that the FPR is below 5 % when the adulteration level is below 13 % and that the FNR is below 5 % when the adulteration level is above 17 %. A recent study (Nichani et al., 2020) followed similar lines in a preliminary method performance characterization study using quantitative decision scores (called D scores in the study). The NTM under consideration was developed in order to distinguish spelt and wheat cultivars.

Validation approach B (Fig. 3, top right) is used when (i) classes are qualitatively delimited, e.g., olive oil from Italy (class α) and Turkey (class β), and (ii) validation is based on decision scores. Two statistical distributions for the decision scores associated with the two classes are shown (as an example). If the decision limit is 2, about 10 % of Italian olive oil samples are misidentified as originating from Turkey (FPR of 10 %). Several validation procedures for this approach and worked-out examples for the calculation of performance characteristics have been described in a recent study (Uhlig et al., 2021). Applying validation approach B was also one of the central themes of another publication (Alewijn et al., 2016), in which the authors described a case study to validate a method to detect organic and conventional eggs (a two-class problem).

Moving to validation approach C (Fig. 3, bottom left). It is used when (i) the classes are delimited by the level of seed oil adulteration in olive oil and (ii) validation is based on y/n decision outcomes. The diagram shows the relative frequencies of samples tested at different adulteration levels along with a probability of detection function for an adulteration limit of 15 %. A statistical model for the probability of detection in connection with collaborative studies of binary test methods is described in (Uhlig et al., 2013).

The probability of identification (POI) approach described in (Labudde & Harnly, 2012) is an example of validation approach C. The method under consideration was developed to distinguish between botanicals with acceptable levels of expected ingredients and those with unacceptable levels. The POI is obtained from the measurement of specific superior test materials (SSTM) and specific inferior test materials (SITM) in different mixing ratios so that the functional dependence on the mixing ratio can be determined.

Finally, validation approach D is used when (i) classes are qualitatively delimited, e.g., olive oil from Italy (class α) and Turkey (class β), and (ii) validation is based on y/n decision outcomes. The example involves testing samples and determining the relative frequencies of samples assigned to class β. The FPR thus obtained is 10 %, and the FNR is 4 %. The procedure described in the USP protocol provides a good example of validation approach D (US Pharmacopeia, 2019).

### 5.4 Benefit for using quantitative decision scores for validation: simulation

**Fig. 4:**
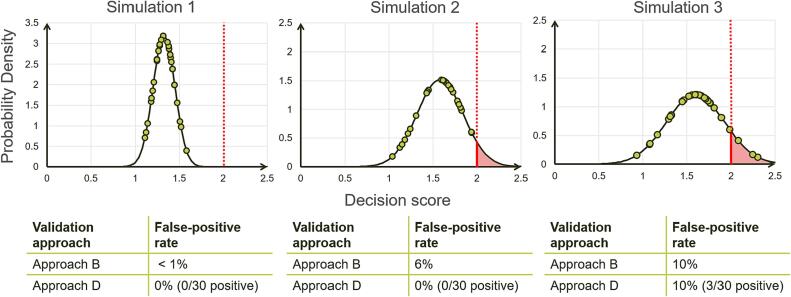
Simulations for illustrating the difference between approaches B and D. Consider the decision limit of 2 (red vertical dotted line) for all three simulations. For each simulation, the green circles represent the decision scores for 30 samples. The region beyond the decision limit of 2 and under the probability density curve (shaded red) is the probability of obtaining a false positive result. (For interpretation of the references to colour in this figure legend, the reader is referred to the web version of this article.)

To demonstrate the implications of using the quantitative decision scores instead of the positive or negative detect (y/n) decision, the following describes a simulated validation study. Fig. 4 illustrates a comparison of the calculated FPR (=1-specificity) according to validation approaches B and D. Three different simulation runs are shown under the assumption that the population of samples can be well described by a normal distribution. We consider 30 validation samples for class α (=negative) (shown as circles) and a decision limit of 2 (shown by a vertical dotted line). For the purpose of this example, the choice of 30 samples is for illustrative purposes only, even though it has been suggested elsewhere (AOAC, 2020e, AOAC, 2020f, AOAC, 2020d). Validation approach B makes use of the quantitative decision scores, whereas approach D is based on the counts of positive and negative results. In the event that the classes can be considered homogeneous and a normal distribution can be used to describe the distribution of the quantitative decision scores, the arithmetic mean and standard deviation of the decision scores can be used to calculate the expected FPR (which is represented by the red shaded area in Fig. 4). For illustrative purposes, we chose a simple example where scores corresponding to only one class are shown (class α, suppose olive oil from Italy) and only compare one performance characteristic, namely, FPR.

Consider the scenario as shown in Fig. 4 simulation 1. The decision scores of all 30 samples are below 2, and therefore the decision is for all samples: class α (=negative). In this situation, approaches B and D will come to the same conclusion. The FPR calculated with approaches B and D are <1 % and 0 % (i.e., 0/30 are positive), respectively. Since the FPR calculations are dependent on the underlying distribution of the data, it is good statistical practice to not claim very small probabilities, and hence we only state that the FPR <1 %. In another scenario, as shown in Fig. 4 Simulation 2, the decision scores of all 30 samples are below 2 and therefore the decision is for samples: class α (=negative). In this situation, approaches B and D will not come to the same conclusion. The FPR according to approach B is 6 % (red shaded area), and according to approach D, it is still 0 %. Alternatively, consider the scenario depicted in Fig. 4 simulation 3. Here, 27 samples were detected as class α (=negative), and 3 samples were detected as not class α (=positive). In this scenario, again, approaches B and D will come to the same conclusion.

For approach D, even if the validation study result is perfect, i.e., FPR = 0 %, it is not apparent whether this result is actually as clear-cut as in simulation 1, or borderline as in simulation 2. One cannot be sure with approach D, even without a single misclassification with 30 samples, that the actual FPR rate is maybe 10 % (see simulation 3). This shows that validation with 30 samples using approach D is very uncertain, and more samples are recommended. Roughly speaking, approach B might lead to better results than approach D by considering the additional distance to the decision limit. It has to be noted, however, that approach B also has statistical uncertainties, like the appropriate choice of the underlying distribution, but these are considerably lower, so that a validation approach with only 30 samples per class seems justified.

The relationship highlighted in comparing validation approaches B and D is also applicable to approaches A and C. Thus, there is considerable benefit in utilizing the decision scores in the validation procedure.

In the case of single lab validation, the decision scores may be used directly. However, for multi-lab validation, standardization of the decision scores across laboratories might be required when different labs have minor differences in the dry lab procedure (e.g., the software or algorithm is different for some labs). In its simplest sense, standardization of scores involves bringing all the decision scores to the same scale so that they can be compared. Certainly, this additional step makes the validation procedure cumbersome. Furthermore, in many cases, the decision scores do not possess a physical interpretation and cannot be traced to a true physical value. Perhaps that is one of the reasons that existing validation procedures (the USP protocol and AOAC SMPRs) only make use of the qualitative y/n outcomes. Altogether, the validation scheme using quantitative scores should make an important contribution to filling the gap in NTM validation. Furthermore, the newly available validation schemes are put together in a framework (as illustrated in Fig. 3) that is not only easy to digest but also helps to choose a suitable scheme with relevant performance characteristics.

## 6 Further considerations for applying NTM validation approaches

When applying a validation approach, experts must take several factors into account. The considerations will influence, among others: (i) what performance characteristics to focus on; (ii) how to determine these performance characteristics; (iii) how to derive performance criteria; and (iv) what data considerations need to be made to derive performance criteria. The various considerations are discussed further below.

### 6.1 Choice of considering the method as screening method or confirmatory method

It has to be distinguished whether the NTM method is to be used for screening purposes or as a confirmatory method. In the case of a screening method, the aim is to identify all samples that could be considered suspect samples. In this case, one will try to minimize the FNR. In the case of a confirmatory method, on the other hand, one will try to prove that the sample is indeed positive. In this case, one will primarily try to minimize the FPR.

### 6.2 Dependence on the measurement platform

NTMs with specialized measurement platforms are becoming more popular, particularly in the research field, due to their high resolution and high analytical sensitivity. Because a particular measurement platform is becoming more widespread, it might be useful to have a validation procedure tailored to it. In this case, the specific nuances of the method can be taken into consideration.

For example, NMR measurements have been used as part of an NTM for testing various types of beverages, such as juice, coffee, wine, beer, and honey (Kuballa et al., 2018). Platform-specific (using the instrument-specific standard operating procedure and, where appropriate, using the reference database of the instrument provider) validation of such proprietary methods allows specific aspects of the platform to be addressed in the validation process. On the other hand, it appears that a platform-independent approach is more appropriate for purposes of official food control. It is to be expected that, as with targeted methods, systematic differences between platforms are unavoidable. The use of different dry lab approaches can also lead to systematic differences in the results. Therefore, the influence of platform-specific effects as well as the influence of dry lab effects must be checked during validation.

### 6.3 Modular or comprehensive validation

Another option is to choose a modular strategy for validating the wet lab and dry lab procedures sequentially. Given the different parts of NTM, a modular validation can help to avoid surprises at the end. It can be performed only if there is evidence that the wet lab and dry lab performances can be considered independent. This can be possible when dealing with certain microbiological or molecular methods (ISO 16140–1:2016, Microbiology of the Food Chain—Method Validation—Part 1: Vocabulary, 2016). However, in most other cases, the final outcome will be affected by variations in the wet lab and dry lab procedures.

In a comprehensive validation, all the steps until the final decision outcome are included. One of the arguments in favor of performing comprehensive validation is that the method outcomes from the dry lab can seldom be considered independent of the measurement results from the wet lab. They are affected by variations in different steps. How these variations in the wet-lab data translate to the final outcome is an important aspect to be examined. Thus, we believe comprehensive validation will be necessary for NTMs.

## 7 Samples for the validation study

Turning to the question of how many and which samples should be used in the validation study, this section details the different criteria that must be fulfilled. Here, it is important to emphasize the distinction between samples used to develop the reference database (or train a machine learning model) and samples used in the validation study. The former is associated with the method development phase, while the latter is associated with the method validation phase (see Section 8). The samples must, first and foremost, be representative of the population of the foods covered by the method. Consider an example of an NTM to detect if rice is basmati or not. In order to validate the method, it is crucial that rice samples be sourced from the Indian subcontinent and not another region (e.g., Italy). This is important as basmati is largely grown in that region. Secondly, the validation samples must be independent and distinct from the ones that are in the reference database. Additionally, it must be ensured (to the best possible extent) that they are not sourced from the same distributor, supplier, farm, location, or processing plant as the samples used to build the reference database.

Next is the question of how many samples are to be tested in the validation study. NTMs can be formulated in a variety of ways, as discussed in 5, 6. The validation approach and the choice of the number of samples depend on several factors, such as: (i) the desired confidence in the results; (ii) whether an NTM is to be employed as a screening method or confirmatory method; (iii) the scope of the NTM; (iv) the testing burden on the laboratory from an economical and practical perspective; (v) the type of statistical study design adopted (conventional or factorial designs); (vi) considerations for matrix effects; and (vii) variations (e.g., seasonal effects) within the respective sample groups or cohorts. Therefore, claims for an exact number of samples required in a validation study for NTM not only need to be grounded in sound statistical theory but also must consider the details of the method to be validated.

However, a few proposals for the number of samples required for method validation have been previously reported. These numbers should be considered only in connection with the described validation scheme and the underlying statistical assumptions. The AOAC SMPRs suggest 30 validation samples for each adulterant (AOAC, 2020a, AOAC, 2020b, AOAC, 2020c). Another recent report states that for a binary NTM, at least 60 samples per class would be required to ensure, with a statistical certainty of 95 %, that there is the inclusion of at least one sample from a subpopulation with a small proportion of 5 % (Uhlig et al., 2021). This can be ensured by using a factorial approach, in which all subclasses or subpopulations resulting from differences in the cultivation, processing, packaging, and delivery of the food are equally taken into account.

## 8 Stages in the development and validation of an NTM

Owing to the complexity involved in the development of an NTM, with so many different components and steps, it can be foreseen that some manner of cooperative method development can alleviate the resource burden. Herein, the effort in the method development stage is distributed among multiple laboratories (or institutions). For instance, a simple split in the effort is made with respect to the wet lab and dry lab development, performed by separate labs (or institutions) respectively. The cooperative method development with multiple labs represents a new paradigm in method development because such an approach has ramifications on the existing procedures of laboratory accreditation and the establishment of an official method. Efforts will be necessary on this front to introduce procedures and protocols. Until then, the conventional way of method development in one lab will likely continue (see Table 1). However, the concept of cooperative method development is gaining traction, especially for NTM development. And we hope this study will instill new insights and spark further work on the validation of collaboratively developed methods.

**Table 1:** Important phases of method development and method validation, to be performed in a conventional or cooperative way.

Method development	Method Validation
*Wet lab + dry lab + reference database*	
Single lab method development	Validation of the method in a single laboratory using a specific set of samples, followed by a multi-lab validation study with a smaller number of samples.
Multi-lab cooperative method development	Multi-lab validation studies

**Fig. 5:**
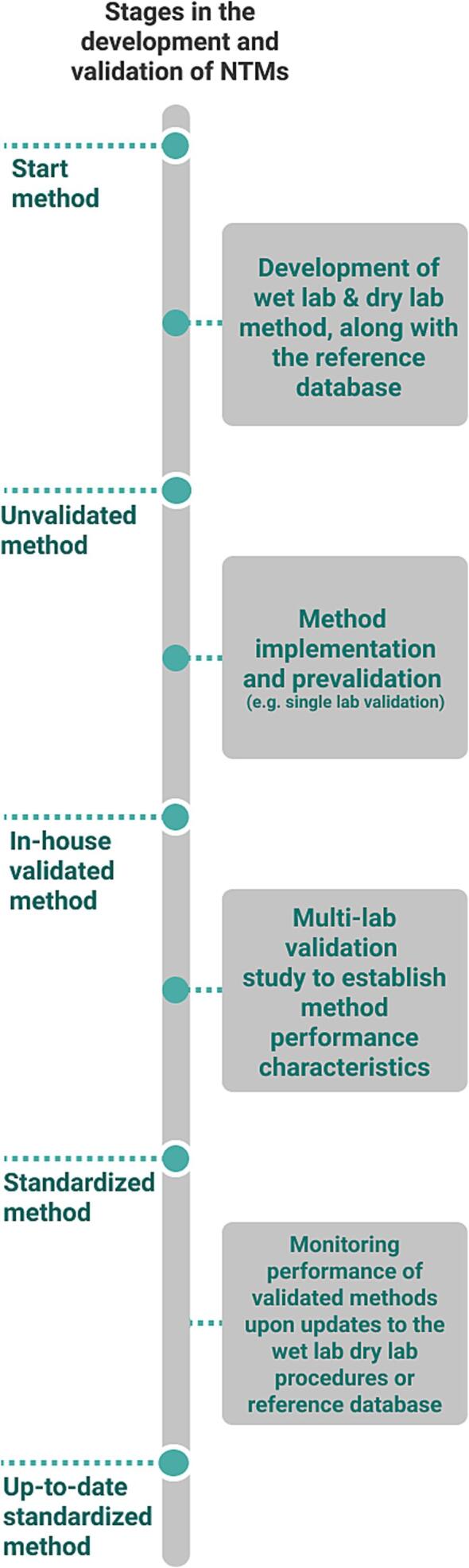
Stages in the development and validation of NTMs.

Fig. 5 illustrates the different stages that are passed through until the standardization of an NTM for food authenticity testing. Once the wet lab and dry lab components of the method are developed, optimized, and perfected for the given method scope, the next natural step is the method validation. The method validation phase has several stages until a method can be adopted for official control. Here, while referring to “method validation,” once again it must be emphasized that it is used in the context of determining method performance characteristics. First, the unvalidated method enters the implementation or prevalidation stage. This stage is referred to as “implementation and prevalidation” because (i) it precedes the most important stage—the multi-lab validation study—and (ii) it allows to further finetune the method as a whole, as results from method implementation can be used to revisit the scope, improve the analytical procedure, or edit spurious entries in the reference database. Typically, after method development, SLV is performed, followed by a method validation interlaboratory test. But if the NTM is developed collaboratively, then single lab validation is inappropriate. SLV would be a suitable option to perform during this stage when a method validation interlaboratory test is followed (see Table 1). Thus, with NTMs, SLV can be performed as part of the prevalidation stage. The exact validation procedure to be adopted and the sample requirements will be based on the discussions made so far in the previous sections.

Another important function of the prevalidation phase is to identify the characteristics of challenging samples, i.e., to determine for which sample types the NTM has particular difficulty finding the correct classification. Interestingly, since challenging is not an inherent characteristic of the sample, a set of challenging samples for one lab may not be challenging for another lab. Evidently, the main outcome of the method implementation and prevalidation stage is to identify such challenging samples. Iterative cycles of development, implementation, and prevalidation can be conducted to improve the method.

The final step in method validation is the multi-lab method validation study, the design for which can be very different: conventional or more efficient factorial designs can be used here (Uhlig & Gowik, 2018). With factorial designs, there is the possibility of reducing the number of labs. Once the multi-laboratory validation study has been performed and the method performance characteristics have been established, the standardization process can be completed and the NTM can be introduced into routine practice.

Even after completion of the method standardization procedure, changes in the underlying reference database, corresponding changes in the dry lab procedure, or extended or amended method objectives can be expected at any time. For instance, the reference sample database can be updated to include a greater number of samples, broadening the scope of food types that can be assessed. Further, the algorithm or the software is updated with a newer version, which might lead to superior discrimination. In these scenarios, the results of the method can deviate drastically. Even though the modifications to the method are aimed at improving it, the validation data may not adequately describe the method's performance. It is therefore necessary to implement a monitoring program for the NTM to control important performance parameters on a regular basis.

## 9 Final discussion and conclusions

Even with a simplified view, NTM terminology can be difficult. And hence, an attempt to deconvolve and disseminate the intricacies has been made in this paper. Our particular focus on the many aspects for NTM validation highlights the fact that devising a validation strategy will require their collective assessment. We discussed how to schematize NTMs for authenticity testing, with the aim of proposing a road map for approaching NTM validation. Ultimately, the intention of an NTM is to classify samples and thereby help in deciding whether a tested sample belongs to a class. The method can be formulated as a single-class, two-class (binary NTM), or multi-class NTM problem. The definition of the underlying classes can be derived qualitatively, e.g., by classifying by geographic origin. Alternatively, they can be derived quantitatively, e.g., by deciding whether the weight percentage of a lower-priced food (adulterant) is below or above a certain threshold. In both cases, the classification is mostly based on a quantitative score value that is compared to a fixed decision limit. The aim of this comparison is either to decide whether a tested sample belongs to a defined group (single-class) or to decide to which of the previously defined groups the sample belongs (multi-class). Validation using a quantitative score value with a fixed decision limit finds its analog in the validation of a measured pollutant concentration with a legal maximum value.

The resulting framework in Section 5 combines and contextualizes both (a) existing concepts and (b) newly proposed concepts for validation. This new framework widens the validation toolbox as it provides the reader with the ability to reason, compare, and select an appropriate validation scheme. The validation procedures discussed so far in this work make a differentiation according to whether the respective qualitative decision results of different laboratories for the validation samples are used in the assessment of the performance of the NTM or whether the respective underlying quantitative score values are also considered for the respective validation samples measured by different laboratories. The number of samples required for validation will have to be determined accordingly; however, some orientation values are discussed. We show the merits of the assessment of the NTM's performance by using this quantitative score value in the validation. A superior validation result can be achieved with a significantly lower validation effort (number of samples and number of laboratories).

The particular challenge now is that the quantitative score value, which forms the basis for the respective classification, cannot be traced back to a specific reference standard. Unlike the determination of sample contents, it cannot be assumed that the quantitative score value fluctuates more or less randomly around a known, true value. When different manufacturers develop NTMs for their respective instrument platforms, the corresponding quantitative score values are not directly comparable. Developing a mathematical-statistical procedure that permits platform-specific quantitative score values to be compared with one another is a mathematically challenging task.

In light of the above discussion, it is essential that NTM development and validation plans be conceived as platform-independent and multi-laboratory from the outset. Individual sub-steps (the development of wet and dry lab methods, method implementation, and prevalidation; see Fig. 5) can be carried out by individual laboratories. Furthermore, all of the NTM's components should be included in the validation planning (see Fig. 1). The contents of the reference database (which can be an existing database if necessary) and the intended decision criteria must be clearly planned and predefined from the beginning. Finally, it must be ensured that the analytical method (wet lab) is suitably standardized (with as few random error components as possible and as few systematic error components as possible) as well as applied comparably by all laboratories in order to generate suitable data for a reference database.

Different statistical models (workflows) are also possible if necessary, and their comparability can be checked during the development and validation of the method. An important difference to targeted methods is that the effort for further quality assurance in the routine is significantly higher. All components of the NTM (see Fig. 1) have to be evaluated (reference database and decision criteria) or quality assured (analytical method and statistical model) at regular intervals. It should be highlighted in this context that it is in the nature of an NTM for questions to change (e.g., slight changes in the underlying classes) or that additions or extensions to the objectives are likely to be the rule rather than the exception. Thus, it is recommended that the (raw) data collected during method development and validation be stored centrally and in a structured form. We believe that embracing and elaborating on the tenets of NTM validation outlined in this paper can guide the development and adoption of suitable validation procedures.

## Declaration of Competing Interest

The authors declare that they have no known competing financial interests or personal relationships that could have appeared to influence the work reported in this paper.

## Data Availability

No data was used for the research described in the article.
